# Removal of the large inverted repeat from the plastid genome reveals gene dosage effects and leads to increased genome copy number

**DOI:** 10.1038/s41477-024-01709-9

**Published:** 2024-05-27

**Authors:** Carolin Krämer, Christian R. Boehm, Jinghan Liu, Michael Kien Yin Ting, Alexander P. Hertle, Joachim Forner, Stephanie Ruf, Mark A. Schöttler, Reimo Zoschke, Ralph Bock

**Affiliations:** Max-Planck-Institut für Molekulare Pflanzenphysiologie, Potsdam-Golm, Germany

**Keywords:** Plant genetics, Plant evolution, Plant genetics, Population genetics

## Abstract

The chloroplast genomes of most plants and algae contain a large inverted repeat (IR) region that separates two single-copy regions and harbours the ribosomal RNA operon. We have addressed the functional importance of the IR region by removing an entire copy of the 25.3-kb IR from the tobacco plastid genome. Using plastid transformation and subsequent selectable marker gene elimination, we precisely excised the IR, thus generating plants with a substantially reduced plastid genome size. We show that the lack of the IR results in a mildly reduced plastid ribosome number, suggesting a gene dosage benefit from the duplicated presence of the ribosomal RNA operon. Moreover, the IR deletion plants contain an increased number of plastid genomes, suggesting that genome copy number is regulated by measuring total plastid DNA content rather than by counting genomes. Together, our findings (1) demonstrate that the IR can enhance the translation capacity of the plastid, (2) reveal the relationship between genome size and genome copy number, and (3) provide a simplified plastid genome structure that will facilitate future synthetic biology applications.

## Abstract (web summary)

Removing the large inverted repeat region from the chloroplast genome revealed a gene dosage benefit for the ribosomal RNA operon. The reduced genome size resulted in increased genome copy numbers and offers potential for synthetic biology.

## Main

**Extended Data Fig. 1:**
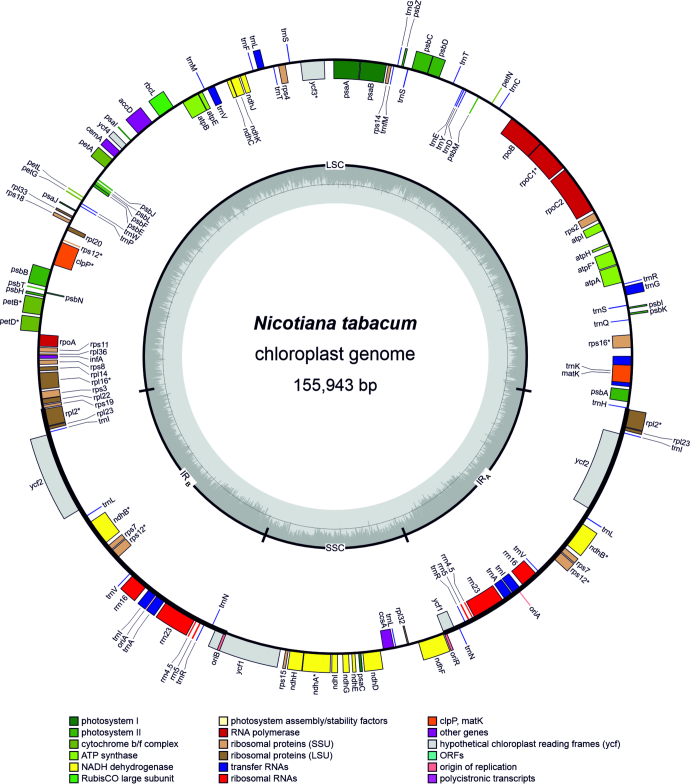
Physical map of the wild-type plastid genome of tobacco (*Nicotiana tabacum*). Genes in the inner circle are transcribed clockwise, genes in the outer circle are transcribed counterclockwise. Intron-containing genes are denoted by asterisks. Genes are color-coded according to functional classes (47). The grey graph in the center indicates the GC content (48). The map was drawn with OGDRAW version 1.3.1 (49).

Plastids (chloroplasts) contain their own genetic information, which is stored in the plastid genome (plastid DNA or ptDNA). The ptDNA maps as a circular genome that, in most embryophytes (land plants), has a tripartite structure. A large single-copy region (LSC) is separated from a small single-copy region (SSC) by a pair of large inverted repeats^1,2^ (IR_A_ and IR_B_). The IRs are identical in sequence and, in a typical seed plant plastid genome, account for approximately a third of the genome size. For example, in the model plant tobacco (*Nicotiana tabacum*), the total size of the ptDNA is 156 kb, and the two IRs together are 50.6 kb (ref. ^3^ and Extended Data Fig. 1). In seed plants, exceptions to the conserved tripartite genome organization are found in a few taxa that secondarily lost one of the IRs for unknown reasons^4,5^. The size of the IR region is variable, and during evolution, both expansions and contractions have occurred. Very large IRs are found, for example, in some species of the family Geraniaceae^6,7^, where the IR size has tripled and become larger than 70 kb. Although the size of the IR region shows substantial variation between species, and the IR borders with the two single-copy regions of the genome can be variable even between closely related species^8^, a nearly universally conserved feature is the presence of the ribosomal RNA (rRNA) operon within the IR^1,2^. The rRNA operon typically encodes the full set of rRNA species contained in the chloroplast ribosome: the 16S rRNA of the small ribosomal subunit and the 23S, 5S and 4.5S rRNAs of the large ribosomal subunit.

The two copies of the IR constantly undergo homologous recombination. Due to their inverted orientation, recombination does not generate subgenomic circles but instead changes the relative orientation of the LSC and SSC (that is, it inverts one single-copy region relative to the other). This phenomenon has been termed flip-flop recombination^9,10^. Its immediate consequence is that the ptDNA occurs in two conformations that are interconvertible and, due to the high efficiency of homologous recombination in plastids, typically co-exist in a 1:1 ratio^9^.

The function of the large IR region in plastid genomes has remained enigmatic. The presence of the rRNA operon within the IR raises the possibility that, by increasing rRNA gene dosage, the IR supports ribosome biogenesis^11^ given that rRNAs account for more than 90% of the cellular RNA content in both prokaryotes and eukaryotes. Interestingly, genes in the IR show substantially slower nucleotide substitution rates than genes in the single-copy regions of the plastid genome^12–16^. This is probably due to copy correction by gene conversion^12,17^ and could be the reason for the presence of highly conserved genes in the IR (rRNA and transfer RNA genes) that need to be protected from mutation. Studies in the few exceptional taxa that lack the IR have revealed elevated mutation rates and increased frequency of genome rearrangements over evolutionary timescales^4,18^, possibly suggesting that the IR may be important for long-term plastid genome stability. However, it is important to note that there are also rearranged plastid genomes that contain an IR (and non-rearranged genomes that lack an IR), suggesting that other factors such as small direct repeats could be more relevant causes of structural instability^5,11,19,20^.

The proposed hypothetical functions of the IR are not necessarily mutually exclusive, and it seems possible that low mutation rates, increased genome stability and elevated gene dosage of IR-resident genes together explain the presence of the IR in plastid genomes. In this work, we have addressed possible functions of the IR by removing one of its two copies from the plastid genome of tobacco (*N. tabacum*). We show that homoplasmic plant lines with a uniform population of IR-deleted plastid genome copies (ΔIR plants) can be obtained. Under standard growth conditions, the ΔIR plants are indistinguishable from wild-type plants. However, they show a developmental reduction in plastid ribosome abundance, indicating that doubled rRNA gene dosage may be one of the benefits linked to IR presence. The substantial reduction in plastid genome size resulting from IR deletion also prompted us to investigate the effect of IR removal on the control of ptDNA content and genome replication. We report that the reduced plastid genome size in the ΔIR plants is compensated by an increase in ptDNA copy number, suggesting that chloroplast genome replication is regulated on the basis of total ptDNA content rather than the number of DNA molecules per organelle.

## Results

### Development of a transplastomic strategy for IR removal

**Fig. 1:**
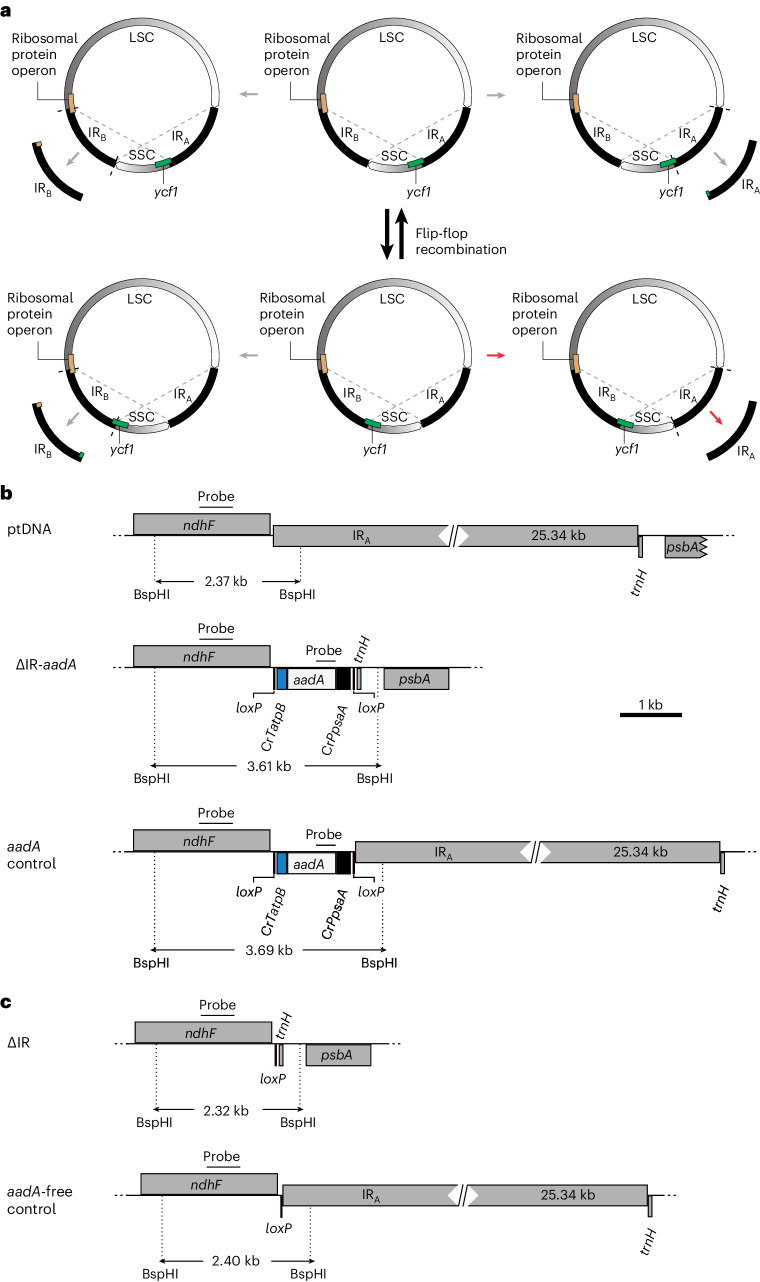
Experimental strategies for IR removal and subsequent selectable marker gene elimination from the plastid genome. **a**, Isoforms of the plastid genome and analysis of the four possible IR deletion scenarios. The ptDNA constantly undergoes flip-flop recombination (shown by dashed lines) resulting in two genome isoforms (depicted in the middle) that differ in the relative orientations of the LSC and SSC. The four theoretical possibilities for IR removal are shown (IR_B_ deletions are on the left; IR_A_ deletions are on the right). Genes spanning the borders between the IRs and the single-copy regions of the genome are indicated to illustrate the expected effects of each deletion scenario. Note that only the genome configuration marked with red arrows retains the integrity of the ribosomal protein operon (brown box) at the LSC–IR border and the essential gene *ycf1* (green box) at the SSC–IR border. See the text for the details. **b**, Physical maps of transformation vectors for the deletion of IR_A_ from the tobacco plastid genome and construction of a control line for neutrality of *aadA* marker insertion. The maps show the targeting region in the tobacco plastid genome (ptDNA) and the engineered regions in the IR deletion lines (ΔIR-*aadA*) and the control lines (*aadA* control). In ∆IR-*aadA* plants, IR_A_ is eliminated and replaced with the *aadA* cassette. The *aadA* control plants harbour the *aadA* cassette in the same site but have two intact IR copies (Methods). Genes above the line are transcribed from left to right; genes below the line are transcribed in the opposite direction. For simplicity, the 25.34-kb IR is indicated by a horizontally broken box (and the genes in the IR are not shown). The *aadA* cassette in both constructs is flanked by *loxP* sites. The expected sizes of DNA fragments in restriction fragment length polymorphism analyses with the restriction enzyme BspHI are given. Hybridization probes (derived from *ndhF* and *aadA*) are indicated by black bars. **c**, Physical maps of the plastid genomes after selectable marker removal mediated by the Cre recombinase. *aadA* excision results in transgene-free plastid genomes that retain only a 34-bp *loxP* scar at the excision site.

The complete deletion of the IR is complicated by the location of two of the borders between the IRs and the single-copy regions in essential genes: the large open reading frame *ycf1* (ref. ^21^) at one of the two IR–SSC borders and a large operon of ribosomal protein genes^3^ at one of the two IR–LSC borders (Fig. 1a and Extended Data Fig. 1). *ycf1* encodes a component of the protein import machinery in the inner membrane of the chloroplast envelope^22^, and its disruption results in the loss of cell viability^21^. In addition to the border regions with the LSC and the SSC, the two different genome conformations resulting from flip-flop recombination^9^ need to be considered for the development of an IR deletion strategy (Fig. 1a).

IR removal can theoretically be achieved with four different deletion constructs that target either IR_A_ or IR_B_ in either of the two genome conformations resulting from flip-flop recombination (Fig. 1a). Three of the four possible deletion strategies are expected to result in lethality, because they lead to the disruption of *ycf1*, the ribosomal protein operon or both (Fig. 1a). The fourth strategy leaves all genes intact by combining them in the single IR copy that is retained (IR_B_ in Fig. 1a).

**Fig. 2:**
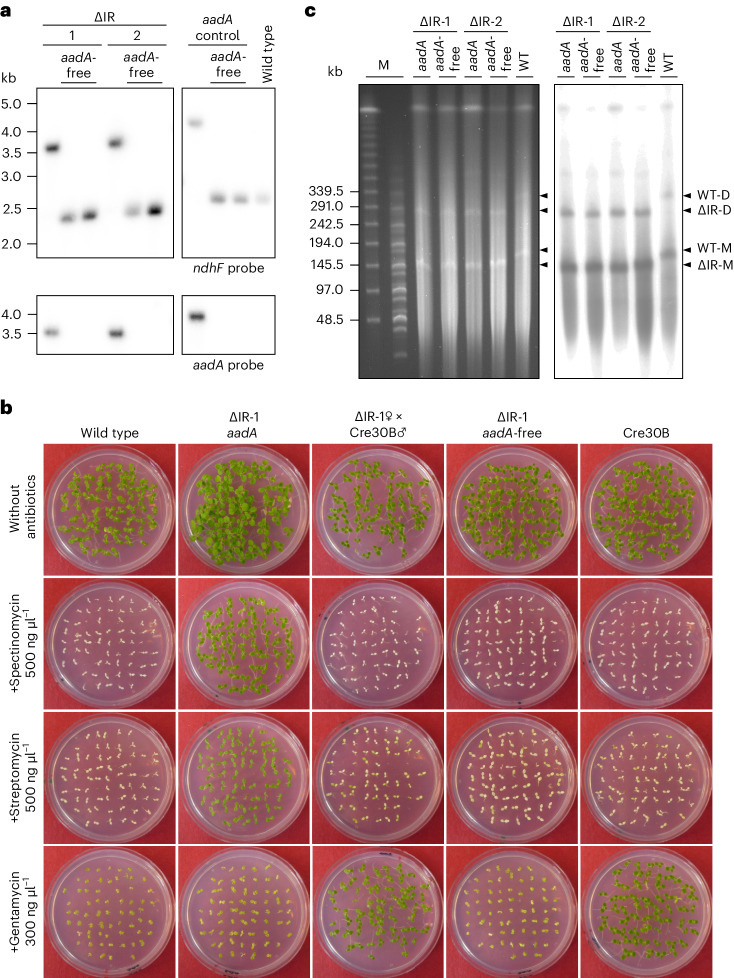
Homoplasmy of transplastomic lines, selectable marker gene elimination and size reduction of the plastid genome resulting from IR deletion. **a**, Restriction fragment length polymorphism analysis of *aadA*-containing and *aadA*-free ∆IR and control lines. Total DNA was digested with BspHI and hybridized to radiolabelled *ndhF* or *aadA* probes (Fig. 1b,c). Homoplasmy is evidenced by the absence of the 2.4-kb *ndhF* fragment diagnostic of the wild-type genome in the *aadA*-containing lines and exclusive detection of the expected fragment sizes (∆IR lines, 3.6 kb; *aadA* control line, 3.7 kb). Successful marker gene elimination is evidenced by a reduction in fragment size that corresponds to the size of the *aadA* cassette (1.3 kb) in the hybridization to the *ndhF* probe (top) and by hybridization to an *aadA*-specific probe (bottom). All samples were run in the same gel (irrelevant lanes were digitally removed). **b**, Seed assay for all steps in the marker elimination process. Seeds were sown on antibiotic-free medium or on media supplemented with spectinomycin, streptomycin or gentamycin. *aadA*-containing transplastomic seedlings are resistant to spectinomycin and streptomycin. The nuclear-transgenic Cre30B line^24^ contains a gentamycin resistance gene. The F_1_ generation is sensitive to spectinomycin and streptomycin but resistant to gentamycin, indicating successful elimination of the *aadA* marker. The F_3_ generation is sensitive to all antibiotics and indistinguishable from the wild-type control (∆IR-1 *aadA*-free). The seedlings were photographed 18 days after sowing. **c**, PFGE showing the size reduction of the plastid genome resulting from IR deletion in the ∆IR mutants. Two *aadA*-containing IR deletion lines and the corresponding *aadA*-free lines were analysed. The ethidium-bromide-stained pulsed-field gel and the Southern blot analysis of the blotted gel by hybridization to a radiolabelled *psaB* probe are shown. The expected sizes for the monomeric form of the plastid genome are 156 kb for the wild type (WT), 132 kb for the *aadA*-containing IR deletion mutants (∆IR *aadA*) and 130.7 kb for the *aadA*-free IR deletion mutants (∆IR *aadA*-free). The monomeric (-M) and dimeric (-D) forms of the plastid genomes are detectable in all lines. M, DNA size markers. The experiments in **a** and **c** were performed once.

On the basis of these considerations, we constructed a chloroplast transformation vector to remove IR_A_ (Fig. 1a,b). The vector contains the appropriate flanking regions taken from the LSC (*psbA* region) and the SSC (*ndhF* region) to enable precise IR excision by homologous recombination. Crossover events in the two flanks replace the IR with the selectable marker gene for plastid transformation, a chimeric *aadA* gene conferring spectinomycin resistance^23^ (Fig. 1b). The *aadA* cassette was equipped with *loxP* sites immediately upstream and downstream to enable post-transformation marker removal by site-specific recombination mediated by the Cre recombinase^24^ (Fig. 2b,c). As a control for neutrality of the marker insertion site in the chloroplast genome, an additional vector was constructed (*aadA* control) that leaves both IRs intact and solely integrates the *aadA* cassette (with its flanking *loxP* sites) into the border between the IR and the SSC (Fig. 1).

### Generation of homoplasmic transplastomic IR-lacking plants

The constructed transformation vectors were loaded onto microparticles and biolistically bombarded into tobacco cells. Selection for spectinomycin resistance conferred by the *aadA* marker gene cassette yielded numerous antibiotic-resistant lines that were subjected to additional rounds of regeneration in the presence of spectinomycin to eliminate residual wild-type copies of the highly polyploid chloroplast genome^25^. Homoplasmy of the transformed plastid genomes was assessed by Southern blotting (Fig. 2a). Two independent homoplasmic ΔIR-*aadA* transplastomic lines and an *aadA* control line (Fig. 1b) were subsequently subjected to selectable marker gene elimination by site-specific recombination. To this end, crosses with a plant line that expresses a plastid-targeted Cre recombinase (linked to a gentamycin resistance gene^24^) were conducted, and the progeny were assayed for loss of the *aadA* marker gene. Homoplasmy of the marker-free plastid genomes was evidenced by both DNA gel blot analysis (Fig. 2a) and inheritance tests (Fig. 2b) that revealed homogeneous loss of spectinomycin resistance in the offspring. Finally, to obtain plant lines that are completely free of transgenes, the gentamycin resistance marker was removed from the nuclear genome by crosses followed by seed assays and PCR tests to identify lines that are no longer resistant to gentamycin (Fig. 2b). A set of PCR assays with primer pairs that distinguish between IR_A_-containing and IR_A_-free genomes confirmed the absence of IR_A_ in the ∆IR plants (Extended Data Fig. 3).

**Extended Data Fig. 2:**
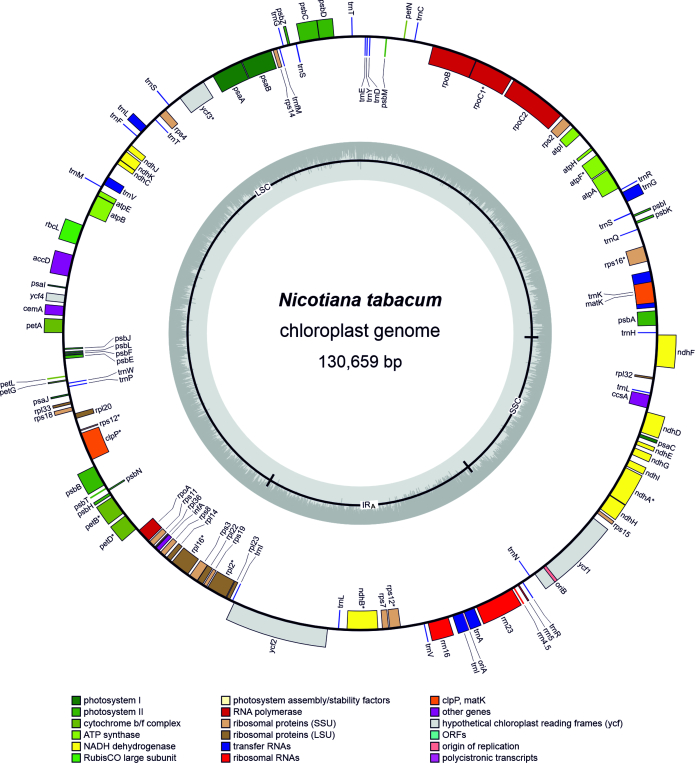
Physical map of the tobacco plastid genome in the IR deletion plants. Note the substantial reduction in genome size compared to the wild-type genome (cf. Extended Data Fig. 1).

**Extended Data Fig. 3:**
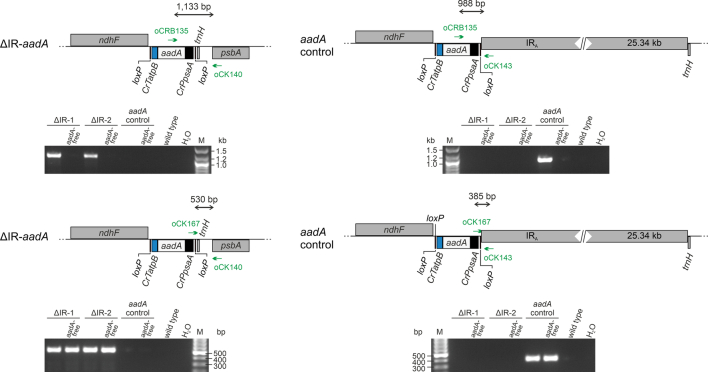
PCR analysis to test for the absence of IR_A_ from the ∆IR plants. Primer pairs flanked the IRA excision site, or the border between SSC and IRA as a control. The binding sites of the PCR primers, along with the expected amplicon sizes, are indicated. The upper panel shows the PCR results for a primer binding within the *aadA* gene, while the bottom panel displays the results for a primer binding within the *loxP* site. The *aadA* control line served as a positive control for amplification of the SSC / IR_A_ border-spanning amplicon. The wild type served as negative control. Note that, due to the abundant presence of promiscuous plastid DNA in the nuclear genome, one of the two PCR primers needs to be derived from either the *aadA* sequence or the *loxP* sequence (that do not occur in the nuclear genome). M, DNA size marker; H_2_O, buffer control (with water instead of DNA added).

To provide ultimate proof of the successful deletion of the IR from the plastid genome, pulsed-field gel electrophoresis (PFGE) of purified chloroplasts was conducted to directly visualize the reduced plastid genome size. The removal of the IR results in a substantially smaller plastid genome of 131 kb (instead of 156 kb in the wild type; Extended Data Figs. 1 and 2)—a size difference that should be readily resolvable by PFGE. Indeed, the chloroplast genomes of the IR deletion lines showed the expected shift in electrophoretic mobility compared with the wild-type ptDNA (Fig. 2c). The size reduction was seen both in the monomeric genomes and in the genome dimers that arise from homologous recombination between two monomers (Fig. 2c).

### Phenotype and physiological characterization of ∆IR lines

**Fig. 3:**
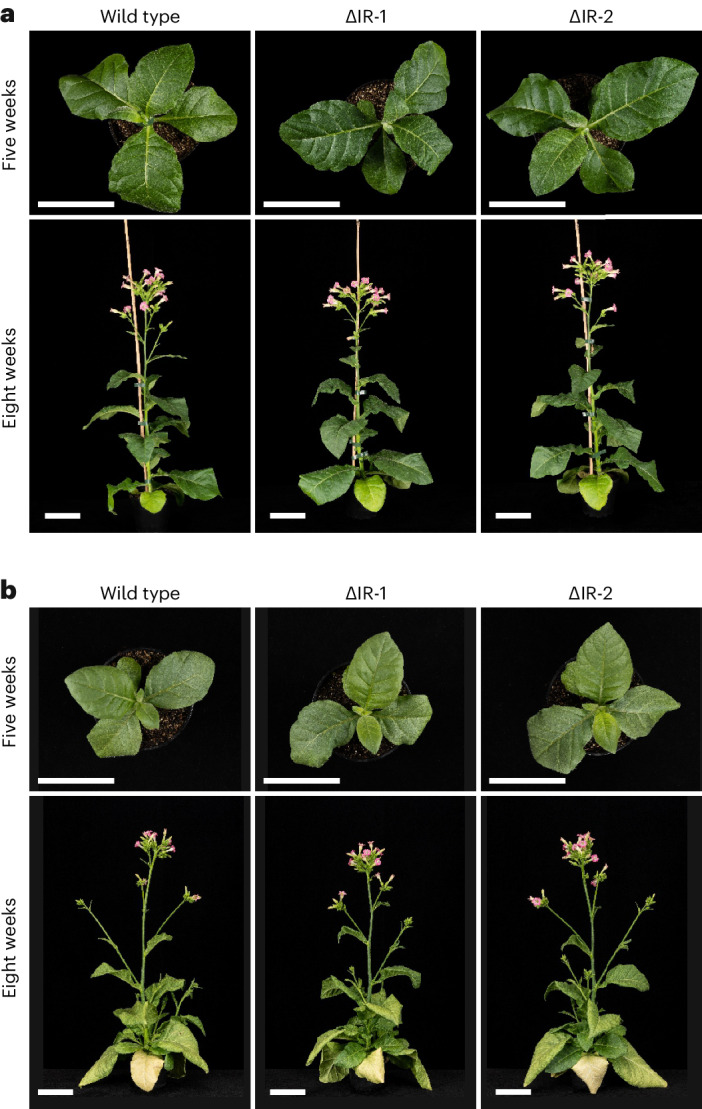
IR deletion plants show a wild-type-like phenotype under different growth conditions. **a**, Plants grown under standard greenhouse conditions (average light intensity, ~300 µmol photons per m^2^ per s). **b**, Plants grown under high-light conditions (1,000 µmol photons per m^2^ per s). Seedlings were transferred to high-light conditions at the age of four weeks after sowing. In both panels, the photos show a representative wild-type plant and two independently generated marker-free IR deletion lines (∆IR-1 and ∆IR-2). The same set of plants is shown at the ages of five and eight weeks. Scale bars, 10 cm.

Having isolated homoplasmic marker-free plants whose chloroplast genomes are identical to that of the wild type except for the absence of one of the 25.3-kb IRs (Extended Data Figs. 1 and 2), we next wanted to determine the phenotypic consequences of the IR removal. To this end, the ΔIR lines were compared to wild-type plants under a variety of different growth conditions (Fig. 3 and Methods). No distinctive growth phenotype was seen under standard greenhouse conditions, and the ΔIR plants were phenotypically indistinguishable from the wild type (Fig. 3a). Exposure to various abiotic stress conditions also did not cause a visible mutant phenotype in the IR deletion plants. Most importantly, chilling stress, an environmental condition that is challenging to both photosynthesis^26,27^ and plastid gene expression activity^28–30^, did not affect the growth or development of the ΔIR lines more severely than those of the wild-type control (Fig. 5a). Finally, to trigger maximum growth rates (which are expected to require optimum performance of photosynthesis and plastid gene expression), plants were exposed to high-light conditions. This also did not lead to a visibly different phenotype (Fig. 3b), suggesting that the absence of the IR does not entail a serious disadvantage under a variety of growth conditions.

**Extended Data Fig. 4:**
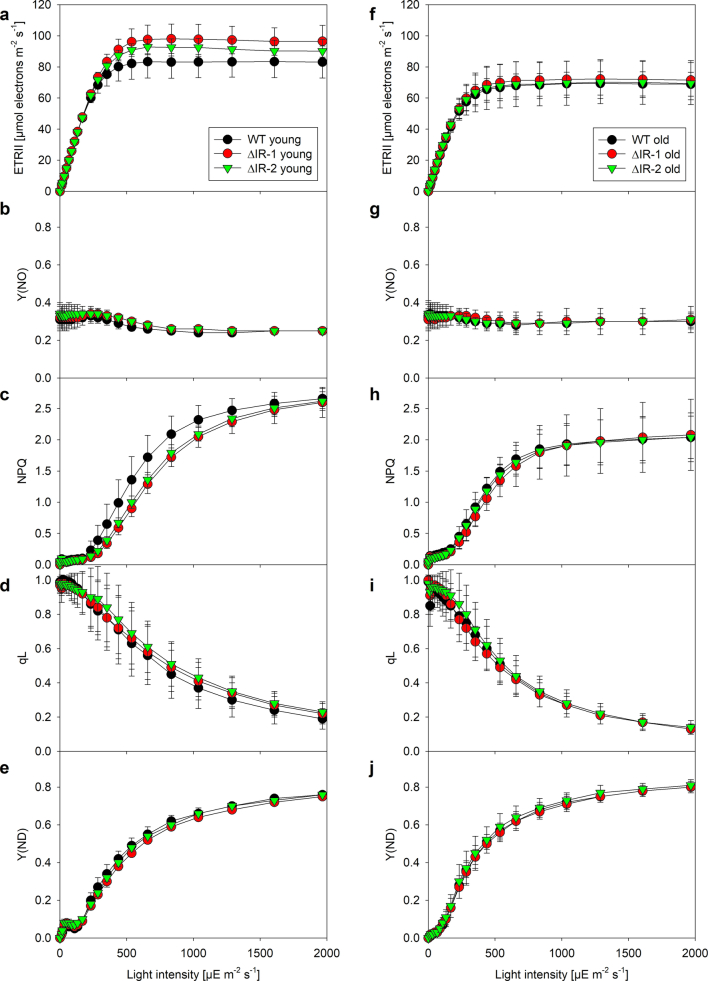
Photosynthetic parameters of high-light acclimated wild-type and ∆IR plants. (**a–e**) Photosynthetic parameters analyzed in the youngest leaves (of ≥17.5 cm length) that fully developed under high-light conditions. (**f–j**) Photosynthetic parameters analyzed in older leaves. The fourth leaf below the young leaf measured in (a-e) was analyzed. Unlike the young leaf, this leaf had already developed prior to the high-light shift. Shown are the chlorophyll-a fluorescence parameters linear electron transport (ETRII; a, f), Y(NO), the non-regulated dissipation of excitation energy in PSII (b, g), the non-photochemical quenching (NPQ; c, h), and the redox state of the PSII acceptor side (qL; d, i). Y(ND), the donor-side limitation of PSI was determined from difference transmittance measurements of P700 (e, j). Data are presented as mean values with error bars indicating the standard deviation (for wild-type plants and ∆IR-2 plants n = 7, for ∆IR-1 plants n = 6; individual data points for the three plant lines are provided as Source Files). Source data

As photosynthesis is the main function of chloroplasts, and most of the plastid-genome-encoded genes are directly or indirectly involved in photosynthesis, we next characterized various photosynthesis-related parameters in high-light-acclimated ΔIR plants. To capture possible developmental differences, all measurements were performed with both young leaves (which developed after the high-light shift) and fully developed leaves (which had already developed prior to the shift; Extended Data Fig. 4). While most parameters were very similar in ΔIR plants and the wild type, a subtle difference was noticed in the light response curves of non-photochemical quenching in young leaves. The ΔIR plants showed a slightly higher capacity of linear electron transport (ETRII), and, accordingly, the induction of photoprotective non-photochemical quenching was shifted to higher light intensities (Extended Data Fig. 4c). Since this effect was seen only in young leaves, it may be related to the biogenesis of the photosynthetic apparatus (which is highly active in developing leaves but largely completed in fully expanded leaves^31,32^). As the biogenesis of the photosynthetic apparatus during leaf development absorbs most of the gene expression capacity of the chloroplast, subtle reductions in plastid gene expression activity often translate into measurable physiological phenotypes only in young leaves^28,29^. On the basis of these observations and considerations, and in view of the presence of the entire rRNA operon in the IR region of the plastid genome, we next turned our attention to the analysis of gene expression in the ΔIR plants.

### IR deletion leads to reduced plastid ribosome numbers

The majority of the genes in the IR of the tobacco plastid genome are involved in translation. In addition to all rRNA genes (16S, 23S, 5S and 4.5S rRNAs), there are four ribosomal protein genes and seven transfer RNA genes contained in the IR (Extended Data Fig. 1). Only two IR-resident genes, the *ndhB* gene (encoding a subunit of the plastid NAD(P)H dehydrogenase complex^33^) and the *ycf2* reading frame (presumably involved in protein import^34^), are not involved in chloroplast translation.

**Fig. 4:**
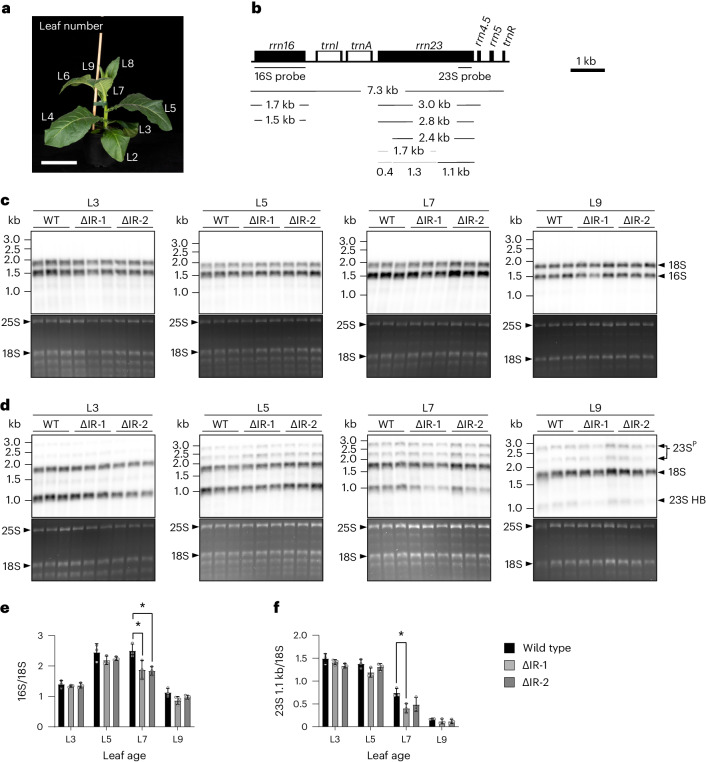
Analysis of plastid ribosome abundance during leaf development. **a**, Developmental stages of tobacco leaves analysed. The leaves were consecutively numbered from the bottom of the plant (excluding the cotyledons), and L3, L5, L7 and L9 were harvested for RNA isolation. A ∆IR-2 plant, grown for six weeks under standard greenhouse conditions, is shown here as a representative example. Scale bar, 10 cm. **b**, Physical map of the plastid rRNA operon, and its transcription and RNA processing products^38,64^. The sizes of major RNA species are given, and the two hybridization probes are indicated by horizontal bars. The 23S rRNA probe used here is derived from the 1.1-kb hidden break (HB) fragment^38^, to simplify the band pattern and facilitate relative quantification. Black bars indicate expected band sizes, grey bars represent 23S rRNA HB fragments and processing products that are not detected by the 23S rRNA probe used here. **c**,**d**, RNA gel blot analyses to quantify plastid rRNAs as a proxy of ribosome abundance. Total cellular RNA was extracted from every other leaf of three biological replicates (individual plants) per plant line (the wild type and two marker-free ∆IR mutant lines, ∆IR-1 and ∆IR-2), separated by denaturing gel electrophoresis, and simultaneously hybridized to a radiolabelled probe for the cytosolic 18S rRNA (serving as a reference for relative quantification) and a radiolabelled probe for either the plastid 16S rRNA (**c**) or 23S rRNA (**d**). The ethidium-bromide-stained gels prior to blotting are shown below each blot as a control for RNA integrity and equal loading. The positions of the cytosolic 25S and 18S rRNAs are marked. The 23S rRNA probe detects, in addition to the 1.1-kb HB product, two precursors (23S^P^) of 2.8 and 2.4 kb (cf. **b**). **e**, Quantification of plastid 16S rRNA abundance relative to the cytosolic 18S rRNA (∆IR-1 L7 *P* = 0.047; ∆IR-2 L7 *P* = 0.0123). **f**, Quantification of plastid 23S rRNA abundance (1.1-kb HB fragment) relative to the cytosolic 18S rRNA (∆IR-1 L7 *P* = 0.0135). The calculations are based on measured band intensities of the hybridized blots. The error bars indicate the standard deviation of the results obtained from the three independent biological replicates. The asterisks denote statistically significant differences (*P* < 0.05, two-sided Student’s *t*-test).

To determine whether the deletion of one of the IR copies affects ribosome biogenesis, we quantified plastid ribosome abundance during leaf development. As rRNA abundance is a reliable proxy of ribosome abundance (in that plastid rRNAs are stable only when incorporated into ribosomal subunits^29,35^), the ratio of chloroplast rRNAs to cytosolic rRNAs can serve as a sensitive indicator of alterations in plastid ribosome numbers^36,37^. We therefore analysed a developmental series of leaves of ΔIR and wild-type plants (Fig. 4a) and determined the ratio of (1) the 16S rRNA in the small subunit of the chloroplast ribosome to the 18S rRNA in the small subunit of the cytosolic ribosome and (2) a fragment of the 23S rRNA in the large subunit of the chloroplast ribosome to the 18S rRNA of the cytosolic ribosome (Fig. 4b–f). The 23S rRNA undergoes post-transcriptional fragmentation (by a process known as hidden break processing^37,38^), resulting in the presence of three fragmentation products in the mature chloroplast ribosome (Fig. 4b).

Both quantifications revealed a reduction of chloroplast ribosome abundance in the ΔIR lines that was particularly pronounced in developing leaves (Fig. 4c–f). While the fully expanded leaves (leaf number 3 (L3)) had very similar numbers of plastid ribosomes, developing leaves (L5, L7 and L9) showed a reduced abundance of plastid ribosomes. The reduction was most pronounced (and statistically significant) in L7 (Fig. 4e,f), but a similar trend was also seen in the slightly older (but also not yet fully expanded) L5 and in the youngest leaf analysed (L9). These results suggest that the reduced gene dosage of IR-encoded ribosomal components limits ribosome biogenesis during leaf development and results in lower numbers of chloroplast ribosomes in our ΔIR plants.

**Fig. 5:**
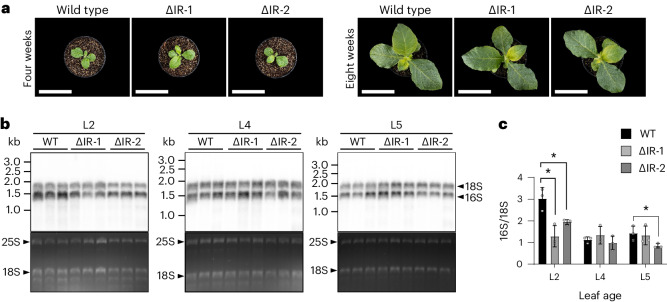
Analysis of plastid ribosome abundance during leaf development under chilling stress. **a**, Phenotypes of plants grown under chilling stress conditions (12 °C, 250 µmol photons per m^2^ per s). Plants were transferred to cold conditions at the age of four weeks. The photographs show the plants at the time of transfer to the cold (four weeks) and at the time of leaf harvest (eight weeks). The experiment was performed once. Scale bars, 10 cm. **b**, RNA blot analyses to quantify rRNAs as a proxy for chloroplast ribosome abundance under cold conditions. L2, L4 and L5 (numbered from the bottom; Fig. 4a) were analysed in three biological replicates. RNA gel blots were simultaneously hybridized to a radiolabelled probe for the cytosolic 18S rRNA (serving as a reference for relative quantification) and a radiolabelled probe for the plastid 16S rRNA (for the details, see Fig. 4). The ethidium-bromide-stained gels prior to blotting are shown below each blot as a control for RNA integrity and equal loading. The bands representing the cytosolic 25S and 18S rRNAs are indicated. **c**, Quantification of plastid 16S rRNA abundance relative to the cytosolic 18S rRNA (∆IR-1 L2 *P* = 0.0132; ∆IR-2 L2 *P* = 0.0228; ∆IR-2 L5 *P* = 0.0450). The calculations are based on measured band intensities of the hybridized blots. The error bars indicate the standard deviation of the results obtained from the three independent biological replicates. The asterisks denote statistically significant differences (*P* < 0.05, two-sided Student’s *t*-test).

As explained above, chilling stress represents a particularly challenging environmental condition that affects both photosynthesis^26^ and plastid gene expression^28,29^. We therefore also analysed plastid ribosome abundance in developing leaves of young plants growing under chilling stress (Fig. 5). These analyses confirmed the reduced plastid ribosome abundance in the ΔIR plants and, in the most strongly affected leaf (L2), revealed an up to 50% lower number of chloroplast ribosomes in the young plants (Fig. 5c).

### Genome-wide plastid transcription and translation analysis

**Fig. 6:**
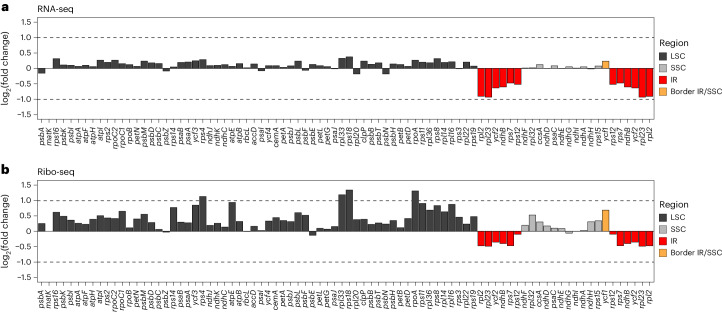
Plastome-wide overview of transcriptional and translational changes in protein-coding genes for ∆IR plants compared with the wild type. **a**,**b**, L5, L6 and L7 from greenhouse-grown plants (Fig. 4a) were harvested and pooled at equal mass for extraction of total RNA and isolation of ribosome footprints. The data summarize results from three biological replicates (that is, three individual plants) for each of the two independent ∆IR lines. Log_2_ fold changes for transcript abundance are shown in **a**, and log_2_ fold changes for ribosome footprint abundance are shown in **b**. Note that the changes measured were significant (false discovery rate, <0.05) at the level of translation only for the genes *rps14*, *rps8* and *rpl16* (Extended Data Fig. 5). Reads from the wild type and the ∆IR mutants were mapped to the same reference genome; therefore, IR reads in the ∆IR mutants are distributed over two IR copies. The data were normalized to all obtained reads for nuclear, chloroplast and mitochondrial genes. For *matK*, no reads were obtained in RNA-seq and ribo-seq experiments. Log_2_ fold changes were obtained from analyses using the R package edgeR.

**Extended Data Fig. 5:**
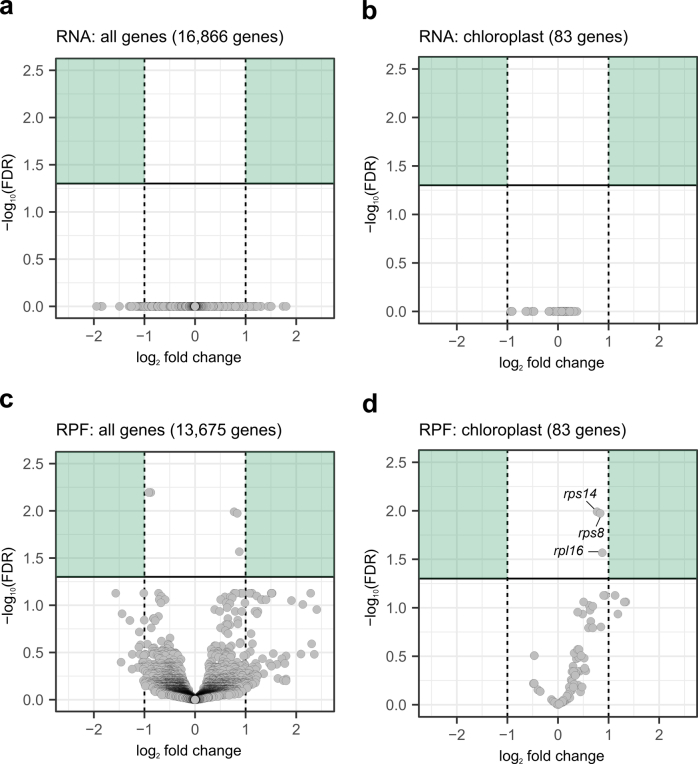
Analysis of transcript accumulation and translational output in wild-type and ∆IR plants. Leaves 5, 6 and 7 from greenhouse-grown plants (cf. Figure 4a) were harvested and pooled at equal mass for extraction of total RNA and isolation of ribosome footprints. The data shown summarize results of three biological replicates (that is, three individual plants) for each of the two independent ∆IR lines. (**a**) Volcano plot of the FDR values versus fold-changes in transcript abundance for all genes analyzed (in the nuclear, plastid and mitochondrial genomes; Supplementary Dataset 1). (**b**) Volcano plot derived from the results in (a), but only showing plastid-encoded protein-coding genes. (**c**) Volcano plot showing the FDR values versus fold-changes in ribosome footprint abundance (RPF: ribosome-protected mRNA fragments) for all genes. (**d**) Volcano plot derived from results in (c), but only showing the plastidencoded protein-coding genes. Significantly changed plastid-encoded genes with an FDR < 0.05 are indicated with their respective gene names. Green colored rectangles mark areas with an FDR < 0.05 and a fold change below 0.5 or above 2.0. Note that none of the detected genes shows a more than twofold up- or down-regulation with statistical significance. Data were normalized to all obtained reads for nuclear, chloroplast and mitochondrial genes. Differential gene expression was analyzed with the R package edgeR.

To examine whether the reduced number of plastid ribosomes in the ΔIR plants can be directly related to gene dosage effects (that is, reduced expression levels of IR-resident genes), we conducted comparative transcript and ribosome profiling experiments with two ΔIR lines and the wild type (Fig. 6). Consistent with the absence of a visibly different phenotype, no strong differences in transcript abundances were detected between the IR deletion and wild-type plants (Extended Data Fig. 5a,b). At the translational level, only three chloroplast genes (*rps14*, *rps8* and *rpl16*) displayed significant differences in ribosome footprint numbers (Extended Data Fig. 5c,d). In general, the measured changes in gene expression were relatively moderate (less than twofold), consistent with the mild alterations in ribosome abundance (Supplementary Data 1). Although most changes in the expression of individual genes were not statistically significant (Extended Data Fig. 5), a plastid-genome-wide view of protein-coding genes revealed a pronounced general trend towards an overall decrease in transcript accumulation and translation of IR-resident genes (Fig. 6a,b). There was also a clear tendency for non-IR genes to display an overall increase in translation in the ΔIR mutants (Fig. 6b), which may be part of a regulatory response to the IR deletion (see below).

Taken together, our analyses of chloroplast ribosome abundance and our comparative studies of mRNA abundances and translational output provide strong evidence for a function of the IR in gene expression. By doubling the gene dosage of key components of the plastid translational apparatus, the IR enhances plastid ribosome biogenesis and contributes to the efficiency of plastid gene expression during leaf development, when the maximum gene expression capacity is needed.

### Control of ptDNA replication by genome size

The generation of plants with a substantially reduced plastid genome size provides unique genetic material to investigate the correlation between genome size and genome copy number. In this regard, two fundamentally different scenarios are theoretically possible. If no correlation between genome size and copy number exists, the copy number of the plastid genome in the IR deletion plants should be identical to that in the wild type. This would imply that plastid genome replication is regulated by the cell ‘measuring’ the number of plastid genome molecules, and if a sufficient number has been reached, DNA replication stops. In the alternative scenario, the cell would measure not plastid genome copy numbers but ptDNA content (and would adjust the DNA amount per organelle to a fixed value). In this scenario, the copy number of the plastid genome should increase in the ΔIR plants proportionally to the reduction in genome size. The size reduction of the plastid genome in the IR deletion lines is approximately 16%—a value that is substantial enough to be able to determine whether or not the cell responds to this reduction by increasing plastid genome copy numbers.

**Fig. 7:**
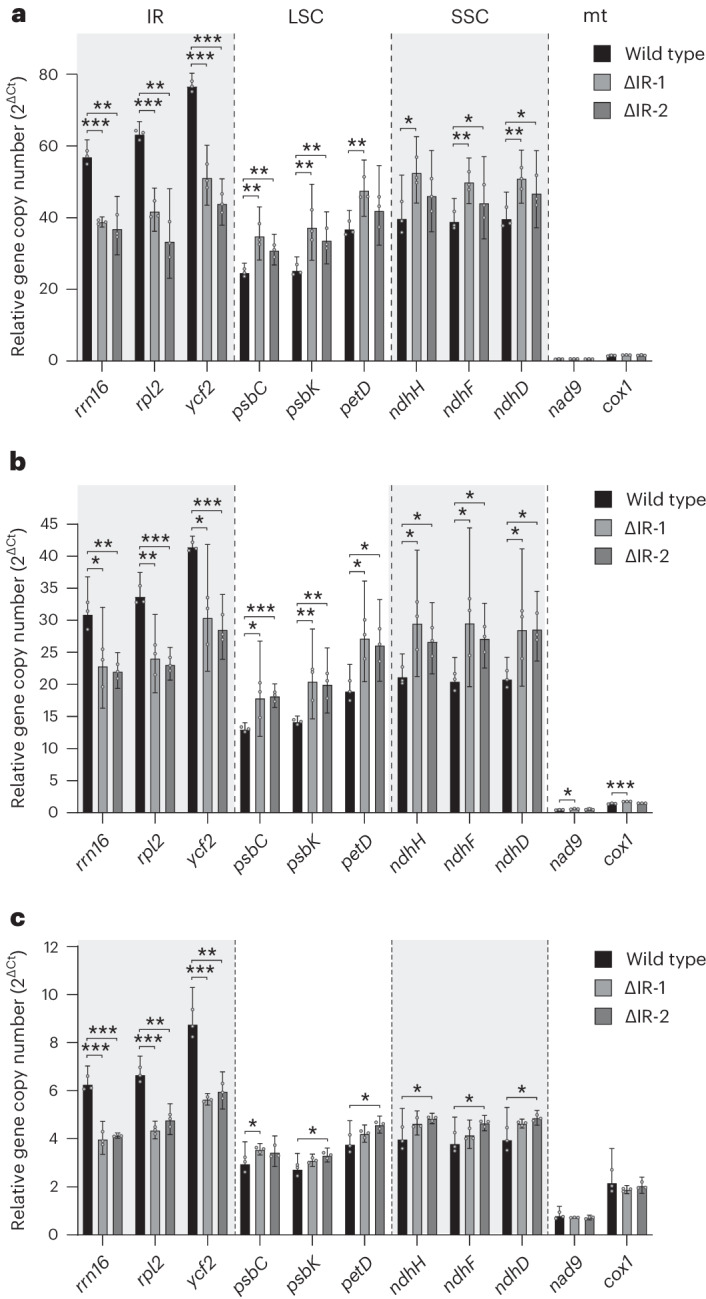
Relative quantification of plastid genome copy numbers in wild-type plants and ∆IR plants by qPCR. **a**–**c**, The analyses were performed with extracted total DNA from pools of five 25-day-old seedlings (**a**); pooled L5, L6 and L7 from greenhouse-grown plants (Fig. 4a) (**b**); and roots from pools of six or seven plants grown in hydroponic culture under aseptic conditions (**c**). The relative gene copy numbers are shown as 2^∆Ct^, where ∆Ct values are Ct (cycle threshold) values normalized to the nuclear genes coding for 18S rRNA and ribosomal protein L25. The error bars indicate the 95% confidence intervals (*n* = 3 biologically independent samples). The locations of the genes analysed are indicated above the plot in **a**. In addition to gene sets in the IR, the LSC and the SSC, two mitochondrial (mt) genes were analysed (which have much lower copy numbers^40^). The determined relative ptDNA copy numbers were analysed using unpaired two-tailed *t*-tests performed on ∆Ct values (*P < 0.05; **P < 0.01; ***P < 0.001; for the corresponding *P* values, see Supplementary Data 2).

To distinguish between the two possible modes of replication control, genome copy numbers were measured using quantitative real-time PCR (qPCR) (Fig. 7 and Methods). To this end, sets of amplicons were derived from (1) three genes residing in the IRs, (2) three genes residing in the LSC, (3) three genes residing in the SSC, (4) two nuclear genes that were used for normalization and (5) two genes in the mitochondrial genome (as additional controls). Genome copy number quantification was performed in seedlings (Fig. 7a), green leaves (Fig. 7b) and roots as a representative non-green tissue (Fig. 7c).

In all three tissue types, genome copy determination in the wild type showed the expected behaviour in that IR-resident plastid genes were present in approximately twice as many copies as LSC and SSC genes. The data also confirmed previous studies revealing that mitochondrial genome copy numbers are far lower than plastid genome copy numbers per cell^39,40^ and that non-photosynthetic plastids contain fewer plastid genome copies than leaf chloroplasts^41^. Estimates in *Arabidopsis* have indicated that, in roots, the copy number of the plastid genome is only one fifth of that in green leaves^41^. The difference determined by our qPCR measurements in tobacco is in the same range (Fig. 7).

**Extended Data Fig. 6:**
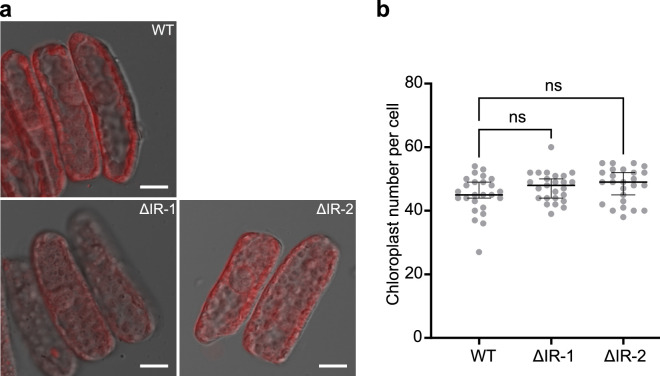
Plastid genome size does not affect chloroplast size and number per cell. (**a**) Confocal microscopic images showing the chlorophyll fluorescence of chloroplasts in mesophyll cells of the wild type (WT) and two ∆IR mutant lines. z-stacks of these images were used for quantification of chloroplast numbers per cell. Scale bars: 10 µm. (**b**) Chloroplast numbers per cell. The black bar indicates the median, shown with the corresponding 95 % confidence interval. Results for each individual cell analyzed are shown as grey dots (n = 25). Chloroplast numbers are not significantly different between the three plant lines analyzed (ns; unpaired two-tailed Student’s t-test, ∆IR-1 *P* = 0.111, ∆IR-2 *P* = 0.072).

When genome copy numbers in the ΔIR plants were determined, a striking pattern was seen. All six amplicons derived from the two single-copy regions of the plastid genome consistently showed a substantial increase in copy number in all three tissue types investigated (Fig. 7). By contrast, all IR-derived amplicons showed a copy-number reduction, which, however, was significantly lower than the expected reduction by 50%. Interestingly, both the increase in copy numbers of the LSC and SSC marker genes and the lower-than-expected reduction in the copy number of the IR marker genes correlated well with the genome size reduction resulting from IR removal, in that the ~16% reduction in genome size led to a similar increase in plastid genome copy numbers in the ΔIR plants (Supplementary Data 2). To exclude the possibility that chloroplast size and/or number per cell are altered in the ΔIR plants, chloroplasts in cells of the wild type and the IR deletion lines were observed by confocal laser-scanning microscopy and counted (Extended Data Fig. 6). No difference in organelle size or number could be detected, strongly suggesting that the observed regulation occurs at the level of genome copy number.

Together, these data strongly suggest that ptDNA replication is not controlled on the basis of genome copy numbers but rather regulated by adjusting the DNA content per plastid to a fixed value.

## Discussion

In this study, we have developed an experimental approach to address the functional relevance of one of the most enigmatic features of plastid genomes: the presence of a large IR region. The IR doubles the gene dosage of a substantial number of chloroplast genes, including the entire rRNA operon, and greatly increases the size of an otherwise extremely streamlined and gene-dense genome^1–3^ (Extended Data Fig. 1). By removing one copy of the IR from the tobacco plastid genome, we reduced genome size by as much as 25.3 kb (Extended Data Figs. 1 and 2) and eliminated flip-flop recombination (Fig. 1a), a salient feature of plastid genomes^9,10^. We have shown that IR deletion and the concomitant genome size reduction are well tolerated by the plant, thus boding well for future efforts to design minimal-size synthetic plastid genomes^42,43^.

Our molecular analyses of the IR deletion plants revealed that plastid gene expression was mildly affected. The effects observed were most pronounced in rapidly growing leaf tissues (Fig. 4) and can largely be attributed to slightly less efficient ribosome biogenesis in the absence of the second copy of the rRNA operon and a set of ribosomal protein genes (Figs. 4–6). Together, our data reported here suggest that, by providing increased gene dosage for key components of the translational apparatus, an important function of the IR lies in the maximization of the capacity of the plastid gene expression machinery. This is needed, for example, in the expansion phase of leaf development, when rapid cell division occurs, necessitating high levels of chloroplast biogenesis and high synthesis rates of the many plastid-encoded components of the photosynthetic apparatus.

While our study has uncovered the functional relevance of the IR to plastid gene expression, the long-term functional consequences of IR removal remain to be determined. It will be interesting to use the plant material generated in this work to grow the IR deletion lines along with the wild type over many generations to be able to assess the long-term stability of the plastid genome. Two aspects seem particularly interesting in this regard: (1) the nucleotide substitution rates in genes that are part of the IR in the wild type and (2) the structural stability of the plastid genome. Since IR-resident genes show substantially lower nucleotide substitution rates than genes residing in the single-copy regions of the genome^13,16^, it would be worthwhile to resequence the plastid genomes in a larger population of IR deletion plants at regular intervals to see whether the expected increase in the nucleotide substitution rate of the remaining copy of the IR can be observed under greenhouse and/or field conditions. Phylogenetic data have also suggested that the loss of the IR can be associated with an increased frequency of genome rearrangements^4,18^. By large-scale genome resequencing over many generations, it may also be possible to observe this effect in real time and thus provide direct evidence for a role of the IR in long-term plastid genome stability.

In addition to revealing the function of the IR in plastid gene expression, our work has provided new insights into the control of plastid genome copy number and the regulation of DNA replication in plastids. Our quantitative analyses of genome copy numbers revealed that in both chloroplasts and non-photosynthetic plastids, IR removal results in substantially increased numbers of plastid genomes (Fig. 7). Remarkably, this increase in copy number is proportional to the decrease in genome size resulting from IR removal. These data demonstrate that chloroplast genome copy number is regulated by measuring the total ptDNA content rather than by counting genome molecules. Our findings also suggest that ptDNA replication is regulated by adjusting the DNA amounts per plastid to a fixed value (which depends on the tissue type; Fig. 7). It will be interesting to identify the factors that are responsible for this regulation. Plastid DNA replication is controlled by nuclear genes^10^, but which gene or genes determine the genome copy number and how this occurs is currently completely unknown. In addition to negative regulatory mechanisms similar to those that control the copy number of bacterial plasmids (at the level of initiation of DNA replication^44^), it seems possible that plastid genome copy number is passively determined by the abundance (and processivity) of the replication enzymes.

Finally, the demonstration that the IR can be eliminated without serious deleterious consequences has implications for future applications in plastid synthetic biology and synthetic genomics^42,43^. Removal of the IR not only makes the plastid genome substantially smaller but also greatly simplifies plastid genome structure. The assembly of synthetic genomes is much more straightforward if sequence duplications can be avoided. As our IR deletion plants are marker-free, they provide an excellent starting point for further size reductions of the genome using transplastomic approaches, including intron removal, deletion of non-essential genes, and refactoring of operons and gene clusters (by what is commonly referred to as top-down synthetic genomics^42^).

In summary, our work reported here (1) demonstrates that the chloroplast genome of seed plants can be substantially reduced in size by excision of the large IR, without entailing noticeable phenotypic consequences; (2) reveals that the IR, via a gene dosage effect, supports high expression rates of the plastid genome; and (3) uncovers how the copy number of plastid genomes per cell is regulated.

## Methods

### Plant material and growth conditions

*Nicotiana tabacum* cultivar Petit Havana plants were grown under sterile conditions on agar-solidified Murashige and Skoog (MS) medium containing 30 g l^−1^ sucrose^45^. The same medium was used for root induction and initial growth of transplastomic lines. For analysis of plant phenotypes and seed production, plants were grown in soil under standard greenhouse conditions (~300 µmol photons per m^2^ per s light intensity under a 16 h light/8 h dark regime with an average day temperature of 25 °C and an average night temperature of 20 °C). For inheritance assays, surface-sterilized seeds were germinated on agar-solidified MS medium supplemented with the appropriate antibiotics. For analysis of root tissue, plants were grown in hydroponic culture under aseptic conditions in liquid MS medium. For growth tests under different environmental conditions, seeds were germinated in soil, and seedlings were grown for four weeks under standard conditions prior to transfer to the stress condition. For growth under high-light conditions, plants were exposed to 1,000 µmol photons per m^2^ per s light intensity (in a 16 h light/8 h dark diurnal cycle). Chilling stress was applied by shifting plants to a day temperature of 12 °C and a night temperature of 8 °C (at 250 µmol photons per m^2^ per s light intensity in a 16 h light/8 h dark diurnal cycle).

### Construction of transformation vectors for IR removal

The construct designed for the deletion of IR_A_ (ΔIR; Fig. 1) contains an *aadA* resistance cassette flanked by 1-kb fragments derived from the regions upstream and downstream of IR_A_ in the targeted chloroplast genome isoform (Fig. 1a). The *aadA* cassette is driven by the *Chlamydomonas reinhardtii* plastid *psaA* promoter (Cr*PpsaA*) and the *atpB* terminator (Cr*TatpB*)^46^. To generate appropriate transplastomic control lines, the same *aadA* cassette was inserted into the junction of IR_A_ and the SSC (Fig. 1). In this construct, the marker gene is flanked by 1-kb fragments of the SSC and IR_A_. In both plastid transformation vectors, the *aadA* cassette is flanked by *loxP* sites to facilitate post-transformation marker gene excision (Fig. 1). All plastid targeting sequences were PCR amplified and purified by agarose gel electrophoresis followed by extraction from excised gel slices (Nucleospin Gel and PCR Clean-up kit; Macherey-Nagel). The vectors are based on pBlueScript II SK (+), and the PCR fragments were inserted between the Acc65I and SacI restriction sites in the polylinker by Gibson assembly reactions (New England Biolabs).

Maps of the plastid genomes of the wild type and the IR deletion lines were generated with OGDRAW v.1.3.1^47–49^.

### Plastid transformation and selection of transplastomic lines

Gold particles were coated with 20 µg of vector DNA and delivered into young, aseptically grown *N. tabacum* cv. Petit Havana leaves via particle bombardment^23^. After biolistic bombardment, the leaves were cut into pieces and placed on regeneration medium containing MS elements with modified vitamins (Duchefa M0245), sucrose (30 g l^−1^), 6-benzylaminopurine (1 mg l^−1^), 1-naphthaleneacetic acid (0.1 mg l^−1^) and agar (5.4 g l^−1^) at pH 5.8. To facilitate selection of transplastomic lines, the medium was supplemented with 500 µg ml^−1^ spectinomycin (Duchefa). Primary spectinomycin-resistant shoots were subjected to additional regeneration rounds to select for homoplasmy. To this end, leaf pieces from antibiotic-resistant lines were transferred to fresh regeneration medium containing 500 µg ml^−1^ spectinomycin. Leaf pieces were additionally cultured on regeneration medium containing 500 µg ml^−1^ streptomycin (Duchefa) to distinguish transplastomic clones from spontaneously arising spectinomycin-resistant mutants^50^. Homoplasmic shoots were transferred to MS medium supplemented with sucrose (3%) and 500 µg ml^−1^ spectinomycin to induce rooting. Rooted shoots were transferred to the greenhouse to produce seeds by selfing.

### Post-transformation excision of the selectable marker gene

The removal of the selectable marker gene via Cre-mediated *loxP* site-specific recombination was conducted as previously described^24,51^. Briefly, marker gene excision was achieved by pollination with a homozygous *Cre*-expressing line that contains a gentamycin resistance marker^24^. The progeny resulting from this cross were selfed, and the next generation was tested for the absence of the *aadA* marker gene and the recombinase gene (linked to the gentamycin resistance gene). To confirm marker gene removal from the plastid genome and outcrossing of the nuclear recombinase cassette, seeds were germinated on MS medium supplemented with spectinomycin (500 µg ml^−1^), streptomycin (500 µg ml^−1^) or gentamycin (300 µg ml^−1^; Fig. 2).

### PFGE

PFGE with isolated chloroplasts was conducted to visualize genome size differences between the plastid genomes of the wild type and the ΔIR mutants. All steps of chloroplast isolation were performed at 4 °C or on ice. About 25 g of young leaves were homogenized in 300 ml of grinding buffer (330 mM sorbitol, 50 mM HEPES, 2 mM EDTA, 1 mM MgCl_2_, 1 mM sodium pyrophosphate tetrabasic decahydrate, 1 mM 2-mercaptoethanol), and the homogenate was filtered through gauze and Miracloth (Merck). The filtrate was centrifuged at 1,700 *g* for 2 min, and the pellet was resuspended in 10 ml of grinding buffer and loaded onto Percoll density gradients (40–80% (v/v) Percoll in grinding buffer, 10% (w/v) PEG6000, 45 mM (+)-sodium l-ascorbate). After centrifugation at 5,600 *g* for 10 min, the intact chloroplasts were collected and washed in wash buffer (350 mM d-sorbitol, 50 mM Tris-HCl pH 7.6). Subsequently, the chloroplasts were collected by centrifugation, resuspended in 300 µl of wash buffer and mixed with 300 µl of 2% (w/v) low-melting-point agarose (InCert agarose, Lonza). The solution was transferred to plug moulds (Bio-Rad), and the solidified plugs were placed in lysis buffer (2% (w/v) Na-laroyl-sarcosin, 0.45 mM EDTA, 10 µg ml^−1^ proteinase K) and incubated at 50 °C for one day, with regular lysis buffer exchange. The plugs were then washed in TE buffer (10 mM Tris-HCl pH 8.0, 1 mM EDTA) and loaded onto a 1% (w/v) agarose gel (Biozym LE, prepared in 0.5× TBE (40 mM Tris-HCl, 50 mM boric acid, 1.25 mM EDTA)), and the wells were sealed with 1% InCert agarose. The DNA was separated in 0.5× TBE using the CHEF Mapper XA System (Bio-Rad). The PFGE conditions were 6 V cm^*−*1^, 14 °C, an angle of 120°, 2.9 to 56 s switch time, 22 h. Finally, the DNA was blotted and analysed by hybridization as described below.

### DNA and RNA gel blot analyses

Total DNA was isolated using a cetyltrimethylammoniumbromide-based DNA extraction method^52^. For Southern blot analyses, samples of 2 µg of DNA were digested with the restriction enzyme BspHI and separated by electrophoresis in a 0.8% (w/v) agarose gel. In preparation for blotting, the gel was incubated in depurination solution (0.25 M HCl) for 15 min, in 0.5 M NaOH for 30 min, in denaturation solution (0.5 M NaOH, 1.5 M NaCl) for 30 min and finally in neutralization solution (1 M Tris, 3 M NaCl, pH 6.5 with HCl) for 15 min.

For total plant RNA extraction, the starch content of the sampled leaf material was reduced by adding 500 µl of SDS buffer (100 mM Tris-HCl pH 8.0, 150 mM NaCl, 50 mM EDTA, 1.5% (w/v) SDS, 2% (v/v) 2-mercaptoethanol) to ~100 mg of ground tissue, followed by incubation on ice for 15 min (ref. ^53^). After centrifugation, the supernatant was mixed with 500 µl of TRIzol reagent (Thermo Fisher Scientific), and RNA extraction was completed following the manufacturer’s instructions. Samples of 500 mg of denatured RNA were separated in a 1.3% (w/v) denaturing agarose gel (containing 16% (v/v) formaldehyde (Sigma), prepared in 1× MOPS buffer (100 mM 3-(*N*-morpholino) propanesulfonic acid, 300 mM NaOAc, 1 mM EDTA, pH 7)).

Electrophoretically separated DNA and RNA samples were transferred to Hybond-XL membranes (GE Healthcare) by overnight capillary blotting in 10× SSC buffer for DNA (1.5 M NaCl, 150 mM trisodium citrate, pH 7.0) or 5× SSC buffer for RNA (0.75 M NaCl, 75 mM trisodium citrate, pH 7). Nucleic acids were covalently bound to the membrane by UV crosslinking (0.12 J cm^−2^; UV-crosslinker BLX-254, Vilber Lourmat).

Probes for DNA and RNA blot analyses were generated by PCR amplification and purified from excised gel slices using the NucleoSpin Gel and PCR Clean‑up kit (Macherey-Nagel). The primers for probe amplification are listed in Supplementary Table 1. The probes were radiolabelled with α[^32^P]dCTP by random priming (GE Healthcare). DNA and RNA blots were hybridized in Church buffer (1% BSA, 1 mM EDTA, 7% SDS, 500 mM Na_2_HPO_4_, pH 7.2) at 65 °C overnight. The membranes were exposed to a storage phosphor screen, and radioactive signals were detected in a Typhoon scanner (GE Healthcare).

Relative ribosome abundances were inferred from hybridization signals of plastid and cytosolic rRNA species on the basis of quantification of signal intensities with Image Lab version 6.1.0 (Bio-Rad).

### Measurements of photosynthetic electron transport

Parameters of photosynthetic electron transport were determined with the modular version of the Dual-PAM-100 instrument (Heinz Walz GmbH) at 22 °C. After 30 min of dark adaptation, the maximum quantum efficiency of PSII in the dark-adapted state (*F*_V_/*F*_M_) was determined. Then, light response curves of the chlorophyll *a* fluorescence parameters linear electron transport, non-photochemical quenching and qL (a measure of the redox state of the PSII acceptor side^54^) were measured^55^. In addition, the donor-side limitation of PSI (Y(ND)) was determined. The measuring times at each actinic light intensity were 150 s under light-limited and 60 s under light-saturated conditions.

### Ribosome profiling and RNA-seq

L5, L6 and L7 (numbered from the bottom of the plant, excluding the cotyledons) were harvested individually from greenhouse-grown, six-week-old tobacco plants and immediately frozen in liquid nitrogen. The frozen leaf material was ground, and equal amounts (w/w) of leaf powder of the three leaves harvested (L5, L6 and L7) were pooled. For each plant line, three biological replicates (that is, three individual plants) were analysed.

Ribosome profiling and rRNA removal were performed as previously described^56^, using 360 mg of pooled leaf tissue. For RNA-seq, an aliquot of total RNA was used as input material for the Zymo-Seq RiboFree total RNA library kit (Zymo), following the manufacturer’s instructions. Data analysis was performed as previously described^56^. In short, genomes and gene annotations were derived from various sources. The tobacco nuclear genome (Edwards assembly) was derived from the Sol Genomics Network (https://solgenomics.net/). The chloroplast and mitochondrial genomes were derived from NCBI (NC_001879.2, and NC_006581.1, respectively). Cutadapt (ref. ^57^) was used to remove adapters and UMIs. Ribo-seq contaminants (rRNA and transfer RNA) were filtered out using STAR aligner^58^ with the following parameters: outFilterMismatchNoverLmax, 0.1; outReadsUnmapped, Fastx. After filtering, the remaining unmapped reads were simultaneously aligned to the three genomes (nuclear, chloroplast and mitochondrial) using STAR aligner with the following parameters: outFilterMismatchNoverLmax, 0.1; alignIntronMax, 8000; outMultimapperOrder, Random. The alignment files were formatted using SAMtools^59^. Mapped reads were counted over all nuclear, chloroplast and mitochondrial genes using FeatureCounts with the following parameters: M, fraction^60^. Differential expression analysis was performed using edgeR, using the default parameters (that is, filtering out genes with less than ten counts^61^). Data visualization was performed in R v.3.5.3 (ref. ^62^) using Tidyverse and ggplot2.

### Relative quantification of plastid genome copy numbers

Total plant DNA samples were used for all qPCR measurements. Assays were performed in technical triplicates for each of the three biological replicates. qPCR measurements were conducted in a LightCycler 480 Real-Time PCR system (Roche), using the associated software for data analysis. The reactions were carried out in a volume of 5 µl containing 2.5 µl of LightCycler 480 SYBR Green I Master (Roche), 500 nM of each primer and ~15 ng of DNA. The PCR conditions were 95 °C for 10 min followed by 45 cycles of 95 °C for 10 s, 60 °C for 10 s and 72 °C for 10 s. For quantification of relative plastid genome copy numbers, a set of three primer pairs was used for each region of the plastid genome (IR, SSC and LSC). In addition, two primer pairs for mitochondrial genes were included. The results were normalized against the *N. tabacum* nuclear *rrn18* (AJ236016.1) gene and the nuclear gene for ribosomal protein L25 (L18908.1). All primers are listed in Supplementary Table 1. Prior to all experiments, the PCR efficiencies were determined for each gene from the slope of DNA serial dilution curves (*E* = (10^(−1/slope)^ − 1) × 100%). Primer pairs with an efficiency of 90–100% were used for the analyses.

### Microscopy

Microscopic analysis of leaf sections was performed as previously described^63^. Briefly, a young leaf was cut into small pieces and submerged in fixation buffer (3.5% glutaraldehyde in 0.1 M phosphate buffer, pH 7.2). After vacuum infiltration, the samples were incubated at 4 °C for 1 h. The fixation buffer was then replaced with 0.1 M Na_2_EDTA, and the samples were kept at 4 °C overnight. The final incubation was done at 60 °C for 2 h and with vigorous shaking. After placing the sample between the microscope slide and coverslip, soft pressure was applied to release and separate individual cells. Image stacks along the *z* axis were captured using a Leica SP8 confocal microscope and the Leica Application Suite X software.

### Reporting summary

Further information on research design is available in the Nature Portfolio Reporting Summary linked to this article.

### Supplementary information

Supplementary Information Supplementary Table 1.

Reporting Summary

Supplementary Data 1 Differential gene expression analysis for RNA-seq and ribo-seq.

Supplementary Data 2 Relative plastid genome copy numbers.

### Source data

Source Data Extended Data Fig. 4 A version of Extended Data Fig. 4 showing individual data points for wild-type plants (*n* = 7), ∆IR-1 plants (*n* = 6) and ∆IR-2 plants (*n* = 7) and the mean values with error bars indicating the standard deviation.

## Data Availability

The data supporting the findings of this study are available within the paper and its Supplementary Information. Sequences from *N. tabacum* are available through Sol Genomics Network (https://solgenomics.net/, Edwards assembly 2017) and NCBI (NC_001879.2 and NC_006581.1). The NGS sequencing data are available under accession number PRJNA1035449 (http://www.ncbi.nlm.nih.gov/bioproject/1035449). Source data are provided with this paper.
