# Supplementary material for: Removal of the large inverted repeat from the plastid genome reveals gene dosage effects and leads to increased genome copy number
Source: Nat Plants. 2024 May 27;10(6):923–35. doi: 10.1038/s41477-024-01709-9 (PMC11208156; doi:10.1038/s41477-024-01709-9)
Supplement: Supplementary file 1 — Supplementary Table 1. [file 41477_2024_1709_MOESM1_ESM.pdf]

nature plants

Supplementary information https://doi.org/10.1038/s41477-024-01709-9

Removal of the large inverted repeat from the plastid genome reveals gene dosage effects and leads to increased genome copy number

In the format provided by the authors and unedited

Table of contents:

Supplementary Table 1

Supplementary Table 1. List of oligonucleotides used in this study.

Name	Sequence (5'-3')	Gene	Usage
oBock104-F	CCCAGAAAGAGGCTGGCCC	psaB	probe for DNA blots
oBock105-R	CCCAAGGGGCGGGAACTGC	psaB	probe for DNA blots
oCRB345-F	GGATCCGGATCCATTATTCATTC AATGG	ndhF	probe for DNA blots
oCRB346-R	CCGATTCATAAGGATAGGAATA AACCGCTTTATGCTC	ndhF	probe for DNA blots
oCRB135-F	CAAATTGCAGTTCGCGCTTAG	aadA	probe for DNA blots
oCRB72-R	GATCGCCGAAGTATCGACTCAA C	aadA	probe for DNA blots
oCK88-F	TGCAACAAACCCCGACTTCT	18S rRNA	probe for RNA blots
oCK89-R	CACCAGACTTGCCCTCCAAT	18S rRNA	probe for RNA blots
oCK38-F	CTCATGGAGAGTTCGATCCTGG	16S rRNA	probe for RNA blots
oCK39-R	AAGGAGGTGATCCAGCCGC	16S rRNA	probe for RNA blots
oCK104-F	AGGGAGCTTGACTGCAAGAC	23S rRNA (HB 1.1 kb)	probe for RNA blots
oCK105-R	TTGGCTACCCAGCGTTTACC	23S rRNA (HB 1.1 kb)	probe for RNA blots
oCK42-F	CATGCCCGAAGTCGTTACCT	16S rRNA	qPCR
oCK43-R	GCACCTTCCAGTACGGCTAC	16S rRNA	qPCR
oCK46-F	ACCAATGCTACGAGCTTCCG	ndhH	qPCR
oCK47-R	GAATCCCCTTCTCGTTGCCA	ndhH	qPCR
oCK52-F	GGCCGGAGCAATGAACCTAT	psbC	qPCR
oCK53-R	AACCTAGAGTAGCTAGGTGGGG	psbC	qPCR
oCK60-F	TTGCGGAGTTGATTATCCCTCTC	nad9	qPCR
oCK61-R	ACTTCGTCTGCACTGGTTTGT	nad9	qPCR
oCK62-F	CGACCCGGCGATCAAATTCT	cox1	qPCR
oCK63-R	ATCATCGCCGGCATAACCAT	cox1	qPCR
oCK68-F	CCGTTAACGAACGAGACCTCA	rrn18	qPCR
oCK69-R	TCAAACTTCCGCGGCCTAAA	rrn18	qPCR
oCK78-F	AGGCTGTCAAGTCAGGATCAAC	L25	qPCR
oCK79-R	ACGAGGGTACTTGGGGTTTCTA	L25	qPCR
oCK108-F	TTCACCCCAACATTCCCCAC	rpl2	qPCR
oCK109-R	GAGGGGAAATCGGCCACATTA	rpl2	qPCR
oCK110-F	TTGGTCCAGGTCATTTCGGG	ycf2	qPCR
oCK111-R	CGTGTCTGGTACTGCATGGT	ycf2	qPCR
oCK114-F	GCTGTTCTCTTATTTGCCGGTG	ndhF	qPCR
oCK115-R	GAGCCGAAATGGGAGTAGGC	ndhF	qPCR
oCK118-F	TTTAGCGGCTTGGCCAGTTA	ndhD	qPCR
oCK119-R	AAAGGTCCCGAGACGAAAATGA	ndhD	qPCR
oCK122-F	GTTCCAATCGCCGTGTAAGC	petD	qPCR
oCK123-R	CTGGATCTTACGGAGCCTGC	petD	qPCR

oCK126-F	GCCCGAGGCCTATGCTTTT	psbK	qPCR
oCK127-R	GAAAACTTACAGCAGCTTGCCA	psbK	qPCR
oCRB135	CAAATTGCAGTTCGCGCTTAG	aadA	PCR
oCK140	CTATCAAGAGGGTGCTATTGCT C	psbA	PCR
oCK143	GAGAAATTCTATGGCTCGGATC	ycf1	PCR
oCK167	CTTCGTATAATGTATGCTATACG AAG	loxP	PCR
