# Supplementary material for: Removal of the large inverted repeat from the plastid genome reveals gene dosage effects and leads to increased genome copy number
Source: Nat Plants. 2024 May 27;10(6):923–35. doi: 10.1038/s41477-024-01709-9 (PMC11208156; doi:10.1038/s41477-024-01709-9)
Supplement: Supplementary file 2 — Reporting Summary [file 41477_2024_1709_MOESM2_ESM.pdf]

nature portfolio

Corresponding author(s): Ralph Bock

Last updated by author(s): Apr 12, 2024

Reporting Summary

Nature Portfolio wishes to improve the reproducibility of the work that we publish. This form provides structure for consistency and transparency in reporting. For further information on Nature Portfolio policies, see our Editorial Policies and the Editorial Policy Checklist.

Statistics

For all statistical analyses, confirm that the following items are present in the figure legend, table legend, main text, or Methods section.

n/a	Confirmed	
☐	☒	The exact sample size (*n*) for each experimental group/condition, given as a discrete number and unit of measurement
☐	☒	A statement on whether measurements were taken from distinct samples or whether the same sample was measured repeatedly
☐	☒	The statistical test(s) used AND whether they are one- or two-sided<br>*Only common tests should be described solely by name; describe more complex techniques in the Methods section.*
☒	☐	A description of all covariates tested
☒	☐	A description of any assumptions or corrections, such as tests of normality and adjustment for multiple comparisons
☐	☒	A full description of the statistical parameters including central tendency (e.g. means) or other basic estimates (e.g. regression coefficient) AND variation (e.g. standard deviation) or associated estimates of uncertainty (e.g. confidence intervals)
☐	☒	For null hypothesis testing, the test statistic (e.g. *F*, *t*, *r*) with confidence intervals, effect sizes, degrees of freedom and *P* value noted<br>*Give P values as exact values whenever suitable.*
☒	☐	For Bayesian analysis, information on the choice of priors and Markov chain Monte Carlo settings
☒	☐	For hierarchical and complex designs, identification of the appropriate level for tests and full reporting of outcomes
☒	☐	Estimates of effect sizes (e.g. Cohen's *d*, Pearson's *r*), indicating how they were calculated

Our web collection on statistics for biologists contains articles on many of the points above.

Software and code

Policy information about availability of computer code

Data collection	The qPCR data was obtained using LightCycler 480 and the corresponding software in version 1.5.0 SP4.
Data analysis	Signal intensities of RNA blots were quantified using Image Lab version 6.1.0 (Bio-Rad).<br>Data was analyzed with Microsoft Excel 2019 MSO v16.0.10377.20023.<br>Data analysis of RNA-seq and ribosome profiling data was performed with cutadapt 2.10 with Python 3.7.6; STAR v2.7.7a; samtools v0.1.19; featureCounts v2.0.1; edgeR v3.38.4; ggplot2 v3.4.2; tidyverse v2.0.0 with R v3.5.3.<br>Chloroplast genomes were visualized using OGDRAW v1.3.1.

For manuscripts utilizing custom algorithms or software that are central to the research but not yet described in published literature, software must be made available to editors and reviewers. We strongly encourage code deposition in a community repository (e.g. GitHub). See the Nature Portfolio guidelines for submitting code & software for further information.

Data

Policy information about availability of data

All manuscripts must include a data availability statement. This statement should provide the following information, where applicable:

- Accession codes, unique identifiers, or web links for publicly available datasets
- A description of any restrictions on data availability
- For clinical datasets or third party data, please ensure that the statement adheres to our policy

The data supporting the findings of this study are available within the paper and its supplementary information files.
Sequences from Nicotiana tabacum are available through Sol Genomics Network (https://solgenomics.net/, Edwards assembly 2017) and NCBI (NC_001879.2 and NC_006581.1)
The NGS sequencing data are available under accession number PRJNA1035449 (http://www.ncbi.nlm.nih.gov/bioproject/1035449).

Research involving human participants, their data, or biological material

Policy information about studies with human participants or human data. See also policy information about sex, gender (identity/presentation), and sexual orientation and race, ethnicity and racism.

Reporting on sex and gender	n/a
Reporting on race, ethnicity, or other socially relevant groupings	n/a
Population characteristics	n/a
Recruitment	n/a
Ethics oversight	n/a

Note that full information on the approval of the study protocol must also be provided in the manuscript.

Field-specific reporting

Please select the one below that is the best fit for your research. If you are not sure, read the appropriate sections before making your selection.

☒ Life sciences ☐ Behavioural & social sciences ☐ Ecological, evolutionary & environmental sciences

For a reference copy of the document with all sections, see nature.com/documents/nr-reporting-summary-flat.pdf

Life sciences study design

All studies must disclose on these points even when the disclosure is negative.

Sample size	No sample size calculations were performed. The analysis of two independently obtained transplastomic lines is standard in the field,as all transplastomic lines are genetically identical due to homologous recombination. All analyses included three independent biological replicates to allow statistical analysis.
Data exclusions	No data were excluded from the analysis.
Replication	The transformation to obtain IR deletion was performed once, successfully generating several independent IR deletion lines. Of these, two lines were used for in-depth analysis. For each condition, three independent biological replicates were analyzed and statistically assessed. In qPCR experiments, each biological replicate was measured in technical triplicate. All attempts of replication are shown in the manuscript, and were successfully performed.
Randomization	For growth under different environmental conditions, plants from different lines were equally distributed in the growth chamber to ensure equal growth conditions. Under all conditions, all genotypes were equally represented, i.e., the same number of plants per transplastomic line and wild type were grown simultaneously under the same growth conditions.
Blinding	Data collection and analysis was not blinded as no subjective parameters were measured that would have benefited from blinding.

Reporting for specific materials, systems and methods

We require information from authors about some types of materials, experimental systems and methods used in many studies. Here, indicate whether each material, system or method listed is relevant to your study. If you are not sure if a list item applies to your research, read the appropriate section before selecting a response.

Materials & experimental systems

n/a	Involved in the study
☒	☐ Antibodies
☒	☐ Eukaryotic cell lines
☒	☐ Palaeontology and archaeology
☒	☐ Animals and other organisms
☒	☐ Clinical data
☒	☐ Dual use research of concern
☐	☒ Plants

Methods

n/a	Involved in the study
☒	☐ ChIP-seq
☒	☐ Flow cytometry
☒	☐ MRI-based neuroimaging

Plants

Seed stocks

Nicotiana tabacum cv. Petit Havana, kept as lab stock for 20 years.

Novel plant genotypes

Transplastomic IR deletion lines were obtained by biolistic transformation via homologous recombination and spectinomycin selection. For post-transformation elimination of the selectable marker gene a previously described homozygous Cre-expressing line was used for crossings (Corneille et al. 2001).
Two independent lines were analyzed in this study, both fom the T4 generation.

Authentication

Homoplasmy of transplastomic lines was confirmed by RFLP analysis. The excision sites of the IR and the selectable marker were verified by sequencing of PCR products.
