# Supplementary figures and images for: Removal of the large inverted repeat from the plastid genome reveals gene dosage effects and leads to increased genome copy number
Source: Nat Plants. 2024 May 27;10(6):923–35. doi: 10.1038/s41477-024-01709-9 (PMC11208156; doi:10.1038/s41477-024-01709-9)

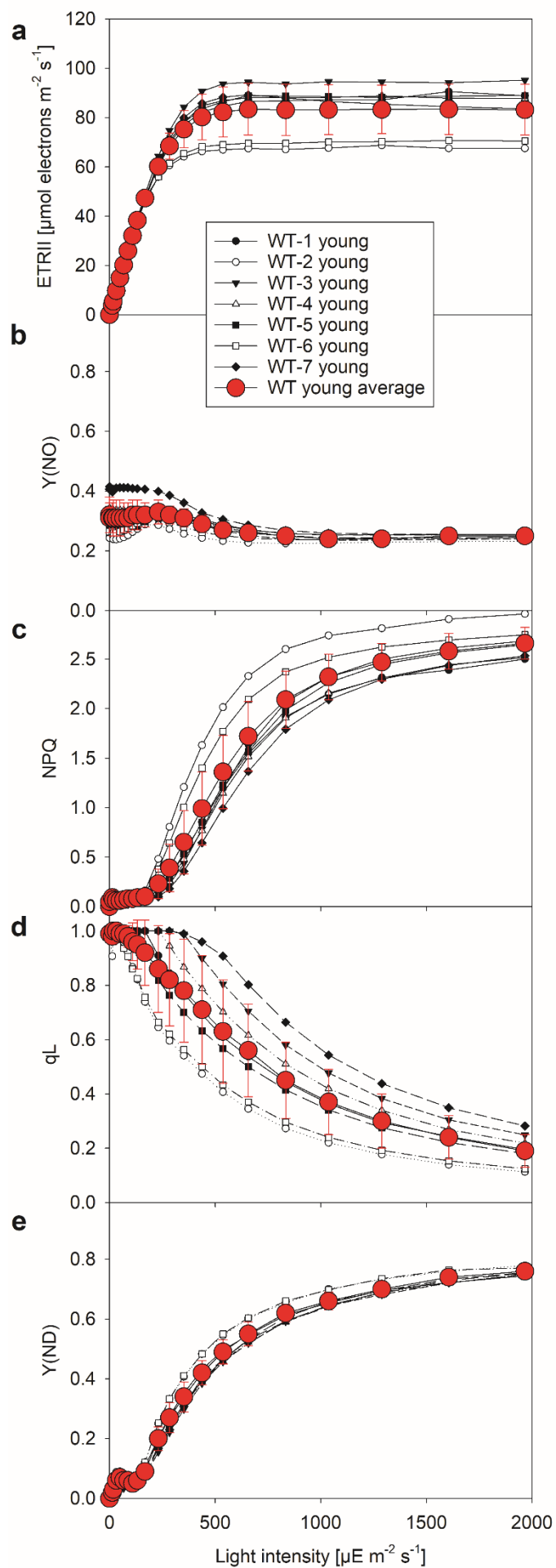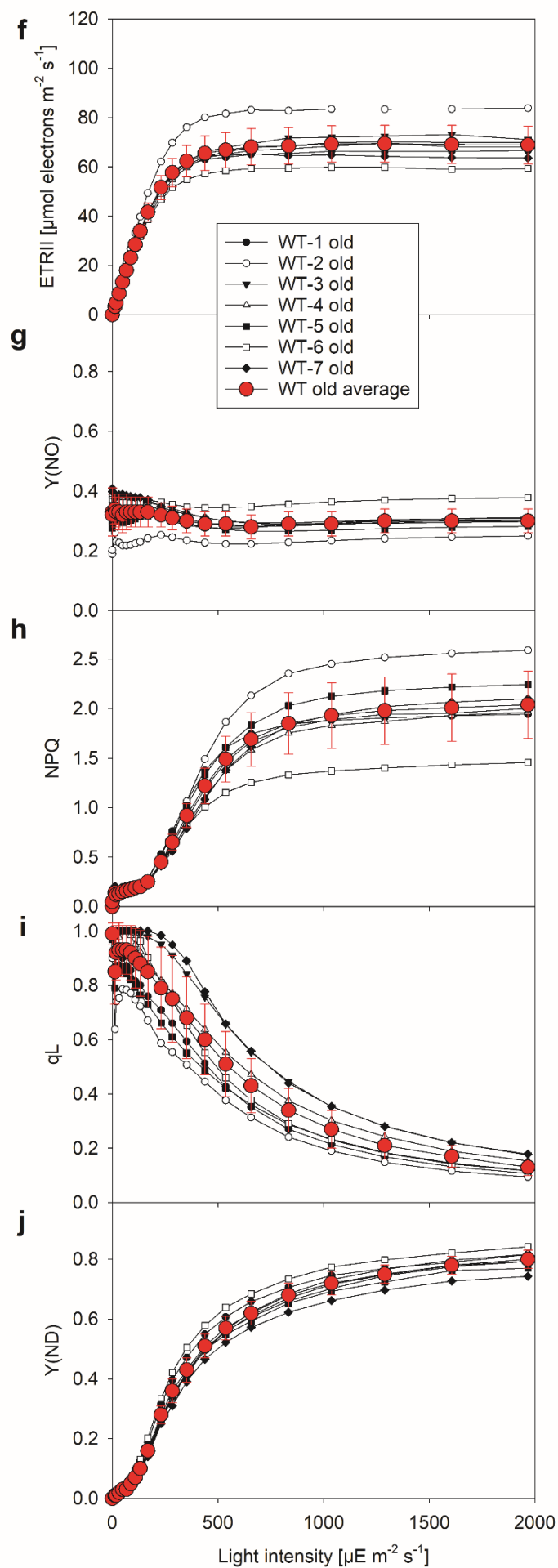

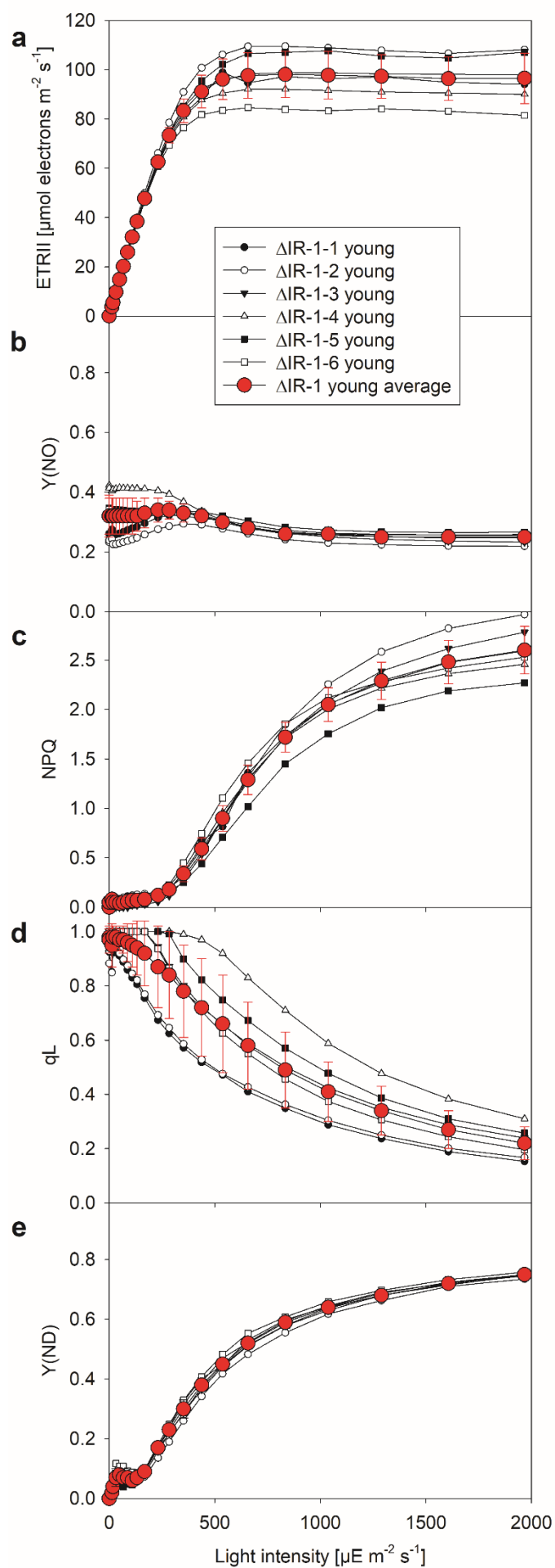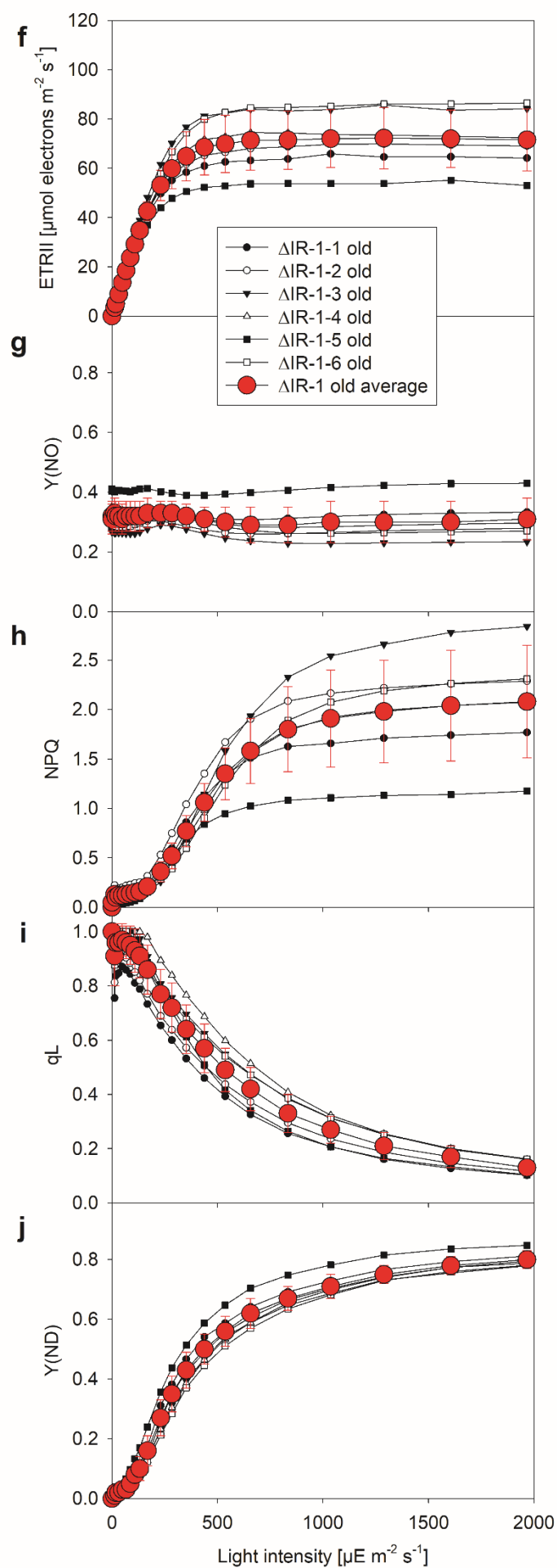

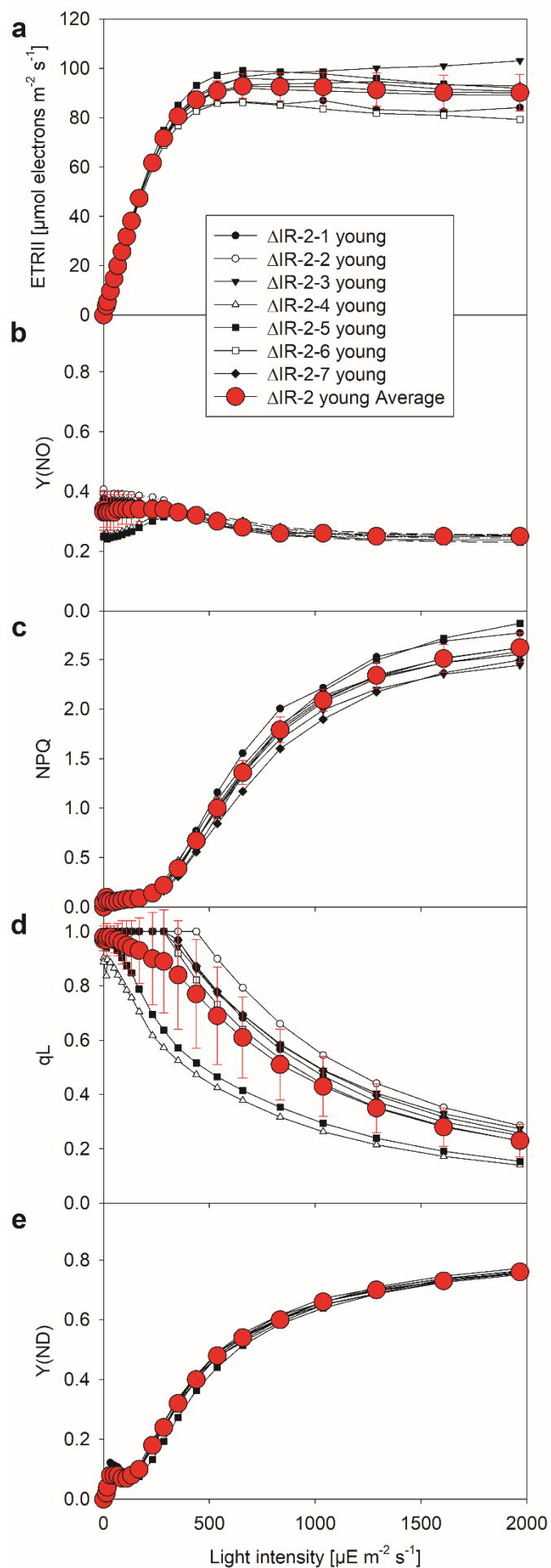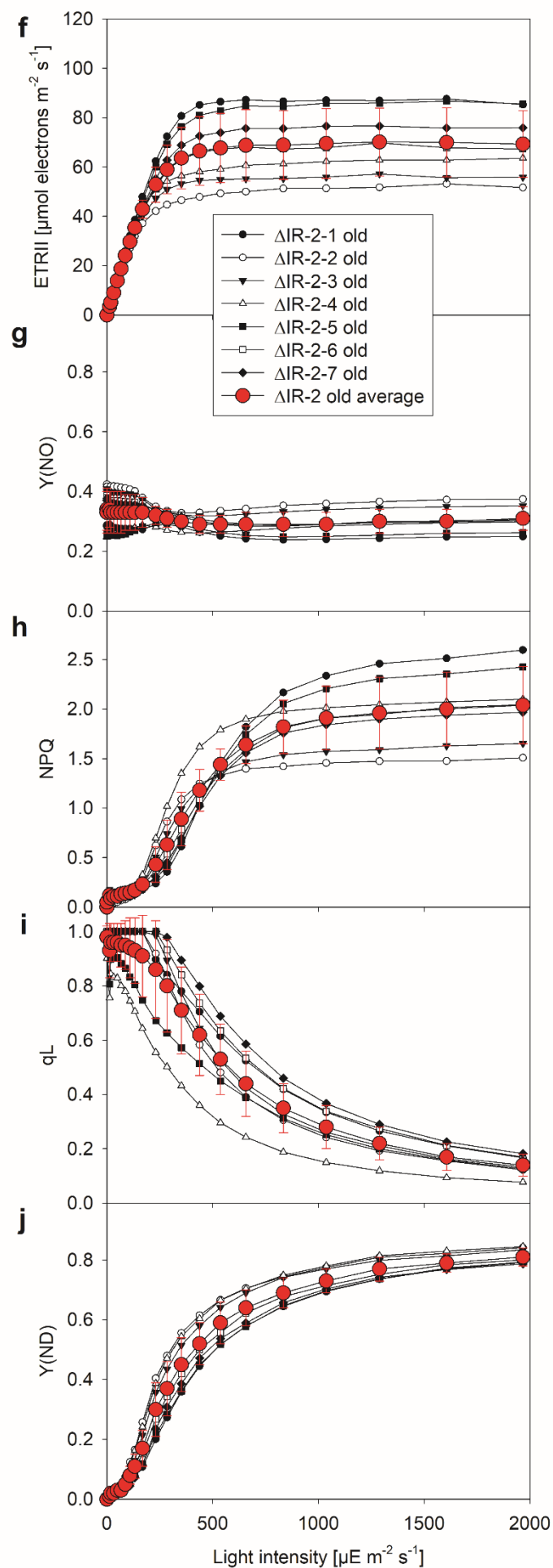

Supplement: Supplementary file 5 — A version of Extended Data Fig. 4 showing individual data points for wild-type plants (n = 7), ∆IR-1 plants (n = 6) and ∆IR-2 plants (n = 7) and the mean values with error bars indicating the standard deviation. [file 41477_2024_1709_MOESM5_ESM.pdf]
